# Subthreshold laser treatment for reticular pseudodrusen secondary to age-related macular degeneration

**DOI:** 10.1038/s41598-021-81810-7

**Published:** 2021-01-26

**Authors:** Giuseppe Querques, Riccardo Sacconi, Francesco Gelormini, Enrico Borrelli, Francesco Prascina, Ilaria Zucchiatti, Lea Querques, Francesco Bandello

**Affiliations:** 1 School of Medicine, Vita-Salute San Raffaele University, Milan, Italy; 2 Division of Head and Neck, Ophthalmology Unit, IRCCS San Raffaele Scientific Institute, Milan, Italy; 3 Department of Ophthalmology, University Vita-Salute, IRCCS Ospedale San Raffaele, Via Olgettina 60, 20132 Milan, Italy

**Keywords:** Eye diseases, Retinal diseases

## Abstract

There is a lack of treatment aimed at the regression of reticular pseudodrusen (RPD) secondary to age-related macular degeneration (AMD). The aim of this prospective, pilot study is to evaluate the safety and short-term efficacy of subthreshold laser treatment (SLT) in patients affected by RPD secondary to dry AMD (dAMD). Twenty eyes of 20 patients (mean age 78.4 ± 6.8 years) with RPD secondary to dAMD were prospectively enrolled. All patients were treated in an extrafoveal area of 1.27 mm^2^ using end-point management yellow subthreshold laser and followed for 3 months. Best-corrected visual acuity was 0.140 ± 0.09 LogMAR at the baseline and no changes were observed during the follow-up (*p* = 0.232). No significant worsening was disclosed before and after the treatment analyzing the macular sensitivity of the treated area (*p* = 0.152). No topical and/or systemic side effects were disclosed during the 3-month follow-up. The distribution among the RPD stages changed after the treatment (*p* < 0.001). In detail, in the treated area, we observed a significant increase in the number of Stage 1 RPD during the follow-up (*p* = 0.002), associated with a significant decrease of Stage 3 RPD (*p* = 0.020). Outer nuclear layer (ONL) thickness analysis showed a significant increase after the treatment associated with RPD regression (*p* = 0.001). End-point management SLT appears a safe treatment for RPD secondary to dAMD, showing short-term safety outcomes. Our results suggest that SLT could be effective in inducing a RPD regression in terms of RPD stage and ONL thickening.

## Introduction

Age-related macular degeneration (AMD) is a progressive chronic disease and the first cause of vision impairment of elderly people in western countries^1,2^. AMD could be classified in two different forms based on the presence of macular neovascularization (MNV): neovascular AMD (nAMD), and non-neovascular or dry AMD (dAMD).

In the last decades, the introduction in the clinical practice of anti-vascular endothelial growth factor (VEGF) injections has changed the natural history of the nAMD, dramatically reducing the visual loss due to the MNV^3^. Conversely, no specific treatment is available to avoid the progression of dAMD into its advanced stage, namely geographic atrophy (GA)^4,5^. Dry AMD is characterized by the progressive loss of the photoreceptors, retinal pigment epithelium (RPE) cells, and choriocapillaris in the affected areas^6–9^. As reported by the Age-Related Eye Disease Study (AREDS) and AREDS2 study, dietary supplementation and general lifestyle modification could reduce the risk of AMD progression in high-risk patients affected by dAMD^10–12^. However, no specific therapies that slow the progression from earlier and asymptomatic stages of the disease into the late stage of dAMD are available. For these reasons, several groups have focused their research on this field, trying to propose new strategies for the treatment of this diffuse and devastating disease.

Reticular pseudodrusen (RPD) are one of the findings characterizing the early and intermediate stages of dAMD, together with drusen and RPE changes^13^. The presence of RPD is associated with a worse visual and anatomical function already from the early stages of the disease^14–17^. Furthermore, several studies have highlighted the role of RPD to accelerate the progression to both forms of late AMD^14,18,19^. For these reasons, a treatment aimed at the regression of RPD could be mandatory in order to prevent the progression to late AMD.

Subthreshold laser is a safe and effective treatment used in the clinical practice in several retinal disorders^20–22^. Although the exact mechanism of action of subthreshold lasers is not completely understood, it has been suggested that it works by targeting, preserving, and “normalizing” the function of the RPE^23^. Since the dysfunction of the RPE has been suggested as the main driving factor in the pathogenesis of RPD, the subthreshold laser could play a crucial role in the treatment of RPD. However, to date, no prospective studies were designed in order to evaluate the safety and efficacy of this treatment in patients affected by RPD. The aim of the current pilot clinical trial is to evaluate the safety and short-term efficacy of the subthreshold laser treatment (SLT) in patients affected by RPD secondary to dAMD.

## Methods

The PASCAL clinical trial is a single-center, non-randomized, pilot study including adults admitted to the Department of Ophthalmology of University Vita-Salute San Raffaele in Milan, Italy, who are suffering from RPD secondary to dAMD.

The trial was conducted in accordance with the Declaration of Helsinki and the study protocol was approved by the Ethics Committee of San Raffaele Hospital (approved on 05/05/2016). The trial was registered on ClinicalTrials.gov (ID NCT02800356, registered on 15/06/2016). All study participants provided written informed consent. The study was conducted in the Medical Retina & Imaging Unit of the Department of Ophthalmology of University Vita-Salute, IRCCS Hospital San Raffaele in Milan, Italy, between June 2016 and September 2019.

The complete list of the inclusion and exclusion criteria is presented in Supplement 1. Briefly, we included patients aged more than 50 years old, with a diagnosis of dAMD and the presence of RPD. We excluded patients with evidence of GA or MNV in the included eye, any prior treatment for AMD in the included eye (aside from antioxidants), and opacities of the ocular media that not permit high-quality imaging examinations.

In cases where both eyes were eligible, the eye with the worse BCVA at baseline was selected as the study eye. If both eyes have the same BCVA, it was recommended to select the right eye as the study eye.

Subjects had the right to withdraw from the study at any time, for any reason, without jeopardizing their medical care.

### Study protocol

The study protocol is summarized in Table 1. After obtaining informed consent and after the screening visit, at the Baseline (Day 0) all patients were evaluated with a complete ophthalmic examination, including Best Corrected Visual Acuity (BCVA) using Snellen charts, slit lamp examination, fundus examination (by indirect ophthalmoscopy) and intraocular pressure measurement (IOP). As imaging protocol, all patients were evaluated using Spectral Domain Optical Coherence Tomography (SD-OCT), fundus autofluorescence (FAF) in the area later treated with laser, microperimetry in a customized area later treated with laser. An extrafoveal area of 1.27 mm^2^ (½ of a disk area, disk area = 2.54 mm^2^) was treated using yellow subthreshold laser (Pascal Synthesis 577 system, Topcon Corporation, Tokyo, Japan). SD-OCT and FAF images were performed using Spectralis HRA + OCT (Heidelberg Engineering, Heidelberg, Germany), whereas microperimetry was performed using MP-1 (Nidek Technologies, Padova, Italy).

**Table 1:** Timing of study assessment.

Visit	Screening	Treatment (baseline)	Follow-up	Follow-up
Point of time	Between − 14 days and day 0	day 0	Month 1	Month 3
Assessment				
Inclusion/exclusion criteria	X			
Informed consent	X			
Demographic data	X			
Medical history	X			
Concurrent medications	X			
Ophthalmic history	X			
BCVA at 4 m prior to dilation	X	X	X	X
Slit lamp examination	X	X	X	X
Fundus examination	X	X	X	X
IOP measurement	X	X	X	X
Macular sensitivity by microperimetry	X	X^a^	X	X
FAF by BAF	X	X	X	X
Structural SD-OCT	X	X	X	X
Subthreshold laser treatment		X		
Adverse events assessment		X	X	X

*BCVA* Best-corrected visual acuity, *IOP* intra-ocular pressure, *FAF* fundus autofluorescence, *BAF* blue autofluorescence, *SD-OCT* spectral-domain optical coherence tomography.

^a^If not executed on screening visit.

All patients were evaluated at 1-month and 3-month follow-up, with a complete ophthalmic examination, including SD-OCT, FAF, and microperimetry.

### Subthreshold laser treatment

**Figure 1:**
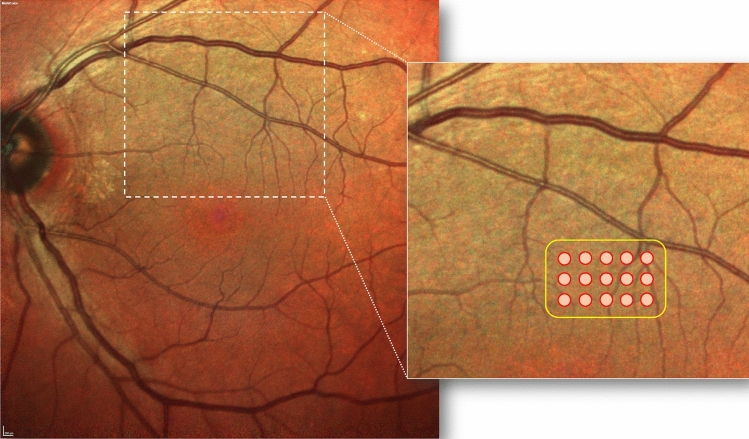
A representative case of an included patient affected by reticular pseudodrusen in the left eye. An extrafoveal area of 1.27 mm^2^ (½ of a disk area, disk area = 2.54 mm^2^) was selected and treated using yellow subthreshold laser (Pascal Synthesis 577 system, Topcon Corporation, Tokyo, Japan). The treatment was performed using the Endpoint Management (~ 30% of the power of the barely visible burn) with a pattern of 5 × 3 spots (area of 1.27 mm^2^).

All the treatments were performed by an expert senior author (GQ). The treatment was performed using the Pascal Synthesis 577 system. During the treatment, the investigator identified the threshold layer within the vascular arcades but outside the central fovea. In detail, the threshold level output power was set to obtain barely visible burn at approximately 200–250 mW using the titration mode. After that, the investigator identified an area inside the vascular arcades affected by RPD. The irradiation was conducted on this area after switching over to Endpoint Management (~ 30% of the power of the barely visible burn) with a pattern of 5 × 3 spots (area of 1.27 mm^2^). Figure 1 shows a representative fundus schema and laser area of the treatment.

### Fundus autofluorescence and spectral domain optical coherence tomography

FAF images were used to detect the possible appearance of GA (defined as a hypo-autofluorescence area) in the treated area during the follow-up. SD-OCT images were used to analyze the outer retinal morphology in the treated area, including evaluation of the stages of RPD at the baseline and during the follow-up as previously reported^24^, and the thickness of the outer nuclear layer (ONL) at the baseline and during the follow-up. Briefly, stage 1 RPD are characterized by diffuse deposition of hyperreflective material between the RPE and the inner/outer segments (IS/OS) boundary; stage 2 is characterized by accumulated material that alters the IS/OS boundary; stage 3 is characterized by thicker and conical appearance of deposited material passing through the IS/OS boundary; stage 4 is characterized by fading of the material due to the reabsorption and migration within the inner retinal layers^24^. The ONL thickness was manually measured in the correspondence of each RPD in the treated area; the mean value was considered for the statistical analysis. All measurements were performed by two independent and experienced readers (FG and EB). The grading of the RPD stage was performed by the same two expert readers. In those cases in which the two graders did not agree on a single consensus result, the final decision was performed by a senior author (GQ).

The retinal thickness and choroidal thickness (ChT) were also recorded. Retinal thickness was assessed in the central 1-mm-diameter circle of ETDRS thickness map [central macular thickness (CMT)] and in the treated area using the Spectralis Software (Heidelberg Eye Explorer, Version 1.10.4.0, Heidelberg, Germany). To achieve good choroidal visualization, enhanced depth imaging (EDI) structural OCT was used in all acquisitions. ChT was assessed in the subfoveal area and in the treated area by manually measuring the distance between Bruch’s membrane and the sclerochoroidal interface to identify the inner and outer boundaries of the choroid, respectively. To better investigate the treated area, a 49 horizontal raster dense linear B-scans, each composed by 16 averaged OCT B-scans (384 A-scans per line) at 30 µm intervals, covering an area of 15 degrees by 5 degrees was performed in each patient at the baseline, at 1-month and 3-month follow-up. All the sections were analyzed in the same place of the retina during the follow-up examinations. In detail, we have obtained the sections in the same place using the follow-up function available on the Spectralis Software (version: 1.10.4.0)^25^.

All the evaluations were also performed in another area of 1.27 mm^2^ with a similar distance from the fovea that did not receive treatment. This area was used as a control area (i.e. area without treatment).

### Microperimetry

Microperimetry was used to assess changes of retinal sensibility in the customized treated area. After training, all subjects underwent scotopic microperimetry examinations of the central retina in the study eye. Prior to testing, pupil dilatation and dark adaptation were performed. The eyes were fully covered with an opaque eye patch, followed by a waiting period of 30 min in a dark room (< 0.1 lx). During the first examination, all patients underwent a fast test only for learning. The anatomical position of the fovea was determined by uploading the combined central infrared reflectance image and horizontal B-scan SD-OCT scan of the Spectralis to the MP-1S software. Using the optic nerve head and the major retinal vessels as landmarks for registration to the fundus real-time image of the MP-1S, test stimuli were placed around the fovea (Goldmann size V, 200 ms, 4-2 strategy, background luminance 0.0032 cd/m^2^, grid centered on the anatomical position of the fovea). Due to the testing under scotopic conditions, the fixation ring was not necessarily centered on the fovea. The following parameters were recorded in each timepoint: overall retinal sensitivity (MS) of the macular area and the MS of the treated area, fixation percentage calculated within the central 2° and 4°^26^.

### Clinical outcome measures

The primary outcome of the PASCAL trial was the safety of treatment measured as retinal sensitivity changes in the treated area 3 months after subthreshold laser treatment (i.e. change in retinal sensitivity).

Prespecified secondary outcomes included:

- Changes in the outer retinal morphology in the treated area using structural OCT during the follow-up;
- Change in mean BCVA during the follow-up;
- Change in the treated area using FAF during the follow-up;
- Adverse and Serious Adverse Events during the follow-up;
- Change in intraocular pressure during the follow-up.

### Statistical analysis

A sample size of 16 eyes has a greater than 80% power to identify a variation of 1.5 decibels in macular sensitivity between pre and post laser treatment assessments, with an estimated standard deviation of the change outcome of 2.0 and an alpha error of 0.05. Allowing an additional 20% of the estimated sample size in order to counter possible withdrawn patients, we estimated that 20 eyes would be required in our series.

All statistical analyses were performed using SPSS Statistics Version 20 (IBM, Armonk, New York, USA). In all patients, BCVA was converted to Logarithm of the Minimum Angle of Resolution (LogMAR) for statistical analysis. Categorical variables were expressed as count and percentage, whereas quantitative variables were expressed as mean ± standard deviation. The agreement between individual measurements from both readers was performed using the intraclass correlation coefficient (ICC; 95% CI). The Gaussian distribution of continuous variables was verified with the Kolmogorov–Smirnov test. Comparisons of BCVA, CMT, subfoveal ChT, retinal and choroidal thickness in the treated area, number of RPD, ONL thickness, IOP, overall MS of the macular area and of the treated area, fixation percentage in the 2° and 4° between different time-points (baseline, 1-month follow-up and 3-month follow-up) were performed using the repeated measures Analysis of Variance (ANOVA) with Bonferroni post-hoc analysis. The comparison between stages of RPD at the baseline and at the end of the follow-up was performed using the Chi-squared test. In all analyses, *p* values < 0.05 were considered statistically significant.

## Results

### Patient demographics and main clinical findings

Twenty eyes of 20 patients (mean age 78.4 ± 6.8 years, median 77.5, range 67–89) fulfilled the criteria of our study and were included. All patients were Caucasians, 15 were females and 5 males. The fellow eye of 15 patients was affected by nAMD, whereas the fellow eye of 5 patients was affected by dAMD. During the 3 months of the study protocol, patients were not treated with any intravitreal injections or other treatments for AMD in the fellow eye.

At the baseline, BCVA was between 20/25 and 20/32 Snellen equivalent (0.140 ± 0.09 LogMAR; median 0.1; range, 0–0.4) and did not shown significant changes at 1-month and 3-month follow-up (BCVA 0.135 ± 0.10 LogMAR; median 0.1; range 0–0.4 and 0.115 ± 0.09 LogMAR; median 0.1; range 0–0.3 at 1-month and 3-month follow-up, respectively) (*p* = 0.232). None of the included patients gained or lost more than 15 letters during the follow-up. IOP did not show significant changes during the follow-up (*p* = 0.267) (Table 2). Furthermore, no significant changes were disclosed analyzing CMT and subfoveal ChT during the follow-up. In detail, CMT was 275 ± 19 µm (median 275, range 229–305) at the baseline, 276 ± 19 µm (median 276, range 237–320) at 1-month follow-up and 273 ± 26 µm (median 273, range 209–324) at 3-month follow-up (*p* = 0.725). Subfoveal ChT at the baseline was 223 ± 110 µm (median 187, range 72–426) and did not show significant changes at 1-month and 3-month follow-up (223 ± 112 µm; median 188; range 77–425 and 221 ± 107 µm; median 184; range 72–433 at 1-month and 3-month follow-up, respectively) (*p* = 0.730) (Table 2).

**Table 2:** Comparisons of anatomical and functional variables between baseline, 1-month follow-up and 3 month follow-up after the treatment.

	Baseline		1-month follow-up		3-month follow-up		
	Mean ± SD	*p* value*	Mean ± SD	*p* value^§^	Mean ± SD	*p* value^§^	*p* value^#^
BCVA, LogMAR	0.14 ± 0.09	0.232	0.135 ± 0.10	1.000	0.115 ± 0.09	0.288	0.311
IOP, mmHg	15.5 ± 2.9	0.267	14.9 ± 2.4	0.827	14.7 ± 2.4	0.300	1.000
CMT, μm	275 ± 19	0.725	276 ± 19	1.000	273 ± 26	1.000	1.000
Subfoveal ChT, μm	223 ± 110	0.730	223 ± 112	1.000	221 ± 107	1.000	1.000
Retinal thickness (treated area), μm	295 ± 26	0.238	294 ± 24	1.000	292 ± 25	0.258	0.847
ChT (treated area), μm	197 ± 88	0.450	195 ± 94	1.000	201 ± 88	1.000	0.613
ONL thickness (treated area), μm	59.30 ± 13.50	0.001	64.75 ± 12.31	0.012	67.75 ± 15.52	< 0.001	0.213
ONL thickness (control area), μm	51.61 ± 10.91	0.199	53.39 ± 12.29	0.645	53.56 ± 12.31	0.204	1.000
MS (macular area), dB	13.99 ± 4.27	0.152	13.05 ± 4.19	0.148	13.65 ± 4.70	1.000	1.000
MS (treated area), dB	12.08 ± 4.65	0.404	11.08 ± 5.06	0.538	11.45 ± 5.70	1.000	1.000
MS (control area), dB	12.01 ± 4.67	0.120	11.02 ± 4.45	0.112	11.69 ± 5.21	1.000	0.910
Fixation percentage (2°), %	42.5 ± 13.2	0.649	39.3 ± 15.1	1.000	40.5 ± 17.3	1.000	1.000
Fixation percentage (4°), %	83.2 ± 12.8	0.342	78.3 ± 15.3	0.550	80.2 ± 11.8	0.969	1.000

*SD* standard deviation, *BCVA* best-corrected visual acuity, *IOP* intraocular pressure, *CMT* central macular thickness, *ChT* choroidal thickness, *ONL* outer nuclear layer, *MS* retinal sensitivity.

*Analysis of Variance (ANOVA) for paired samples.

^§^Comparison with baseline using ANOVA for paired samples with Bonferroni post-hoc analysis.

^#^Comparison with 1-month follow-up using ANOVA for paired samples with Bonferroni post-hoc anaysis.

### Functional changes in the treated area

**Figure 2:**
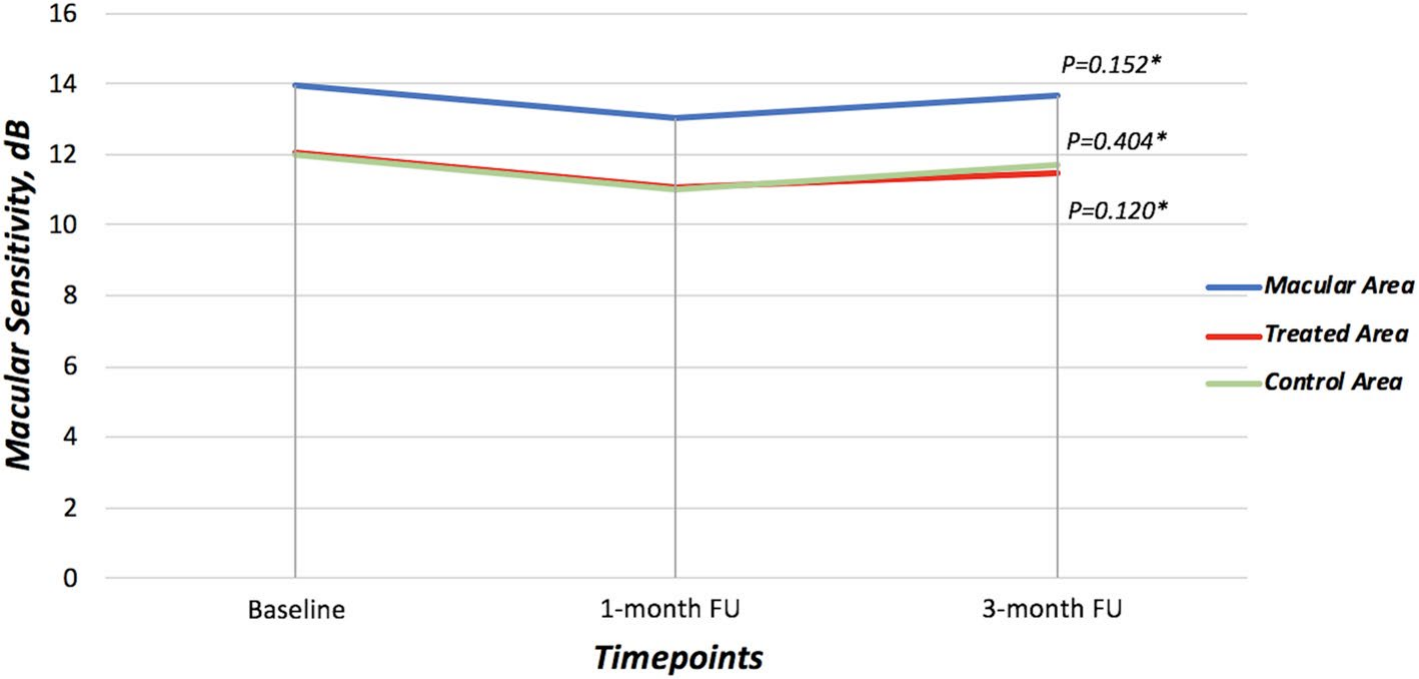
Results of microperimetry in terms of macular sensitivity of the whole macular area, of the treated area, and of the control area during the follow-up. *Analysis of Variance (ANOVA) for paired samples.

Assessment of retinal sensitivity was performed using microperimetry. No significant changes were disclosed before and after the treatment analyzing the overall MS of the macular area and the MS of the treated area. Of note, the overall MS was 13.99 ± 4.27 dB at the baseline and did not significantly change during the follow-up (*p* = 0.152) (Table 2, Fig. 2). Analyzing the mean MS of only the treated area, it was 12.08 ± 4.65 dB at the baseline, 11.08 ± 5.06 dB at 1-month follow-up, and 11.45 ± 5.70 dB at 3-month follow-up (*p* = 0.404). Furthermore, the mean MS of only the control area was 12.01 ± 4.67 dB at the baseline, 11.02 ± 4.45 dB at 1-month follow-up, and 11.69 ± 5.21 at 3-month follow-up (*p* = 0.120) (Table 2, Fig. 2).

Mean fixation percentage calculated within the central 2° (centered on the fovea) was 42.5 ± 13.2% at the baseline and did not significantly change during the follow-up (*p* = 0.649); also mean fixation percentage calculated within the central 4° (centered on the fovea) did not show significant changes during the follow-up (*p* = 0.342) (Table 2).

### Anatomical changes in the treated area

**Figure 3:**
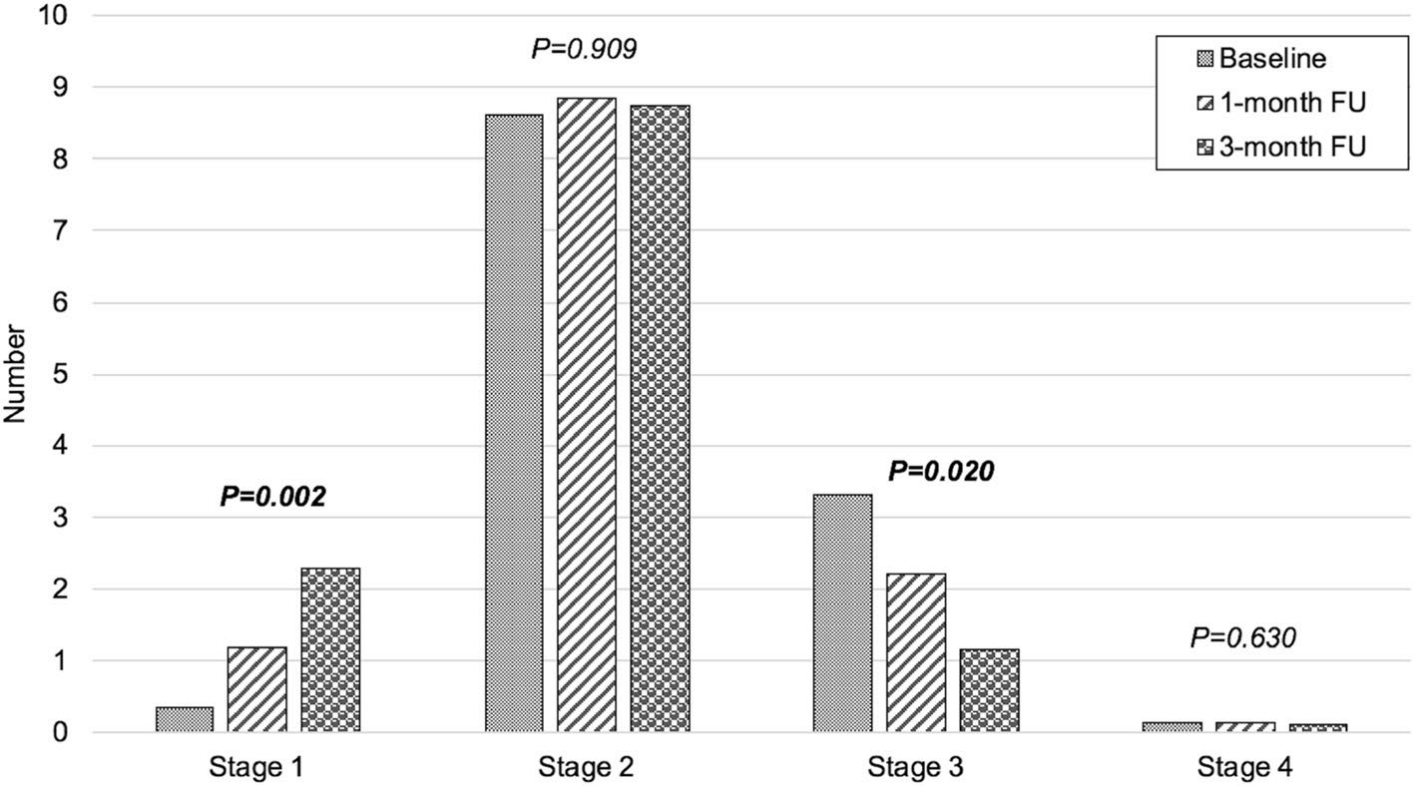
Number of reticular pseudodrusen (RPD) in the treated area according to the stage before and after subthreshold laser treatment. Stage 1 RPD significantly increased after the subthreshold laser treatment (first histogram) due to the significant regression of stage 3 RPD (third histogram). No significant differences were disclosed after the treatment in stage 2 and stage 4 RPD (second and fourth histograms, respectively).

An area of 1.27 mm^2^ was treated in all patients. The area was located at a mean distance of 1904 ± 544 µm (median 1811.5, range 1127–3000) from the fovea. Using structural OCT, we identified a mean of 12.4 ± 6.2 RPD (median 11.5, range 3–23) inside the treated area. The distribution of RPD among the RPD stages changed after the treatment (*p* < 0.001). At the baseline, 0.35 ± 0.59 RPD were classified as Stage 1, 8.60 ± 4.79 as Stage 2, 3.30 ± 4.35 as Stage 3, and 0.15 ± 0.49 as Stage 4. At 1-month follow-up, 1.20 ± 1.70 RPD were classified as Stage 1, 8.85 ± 5.20 as Stage 2, 2.20 ± 3.98 as Stage 3, and 0.15 ± 0.49 as Stage 4. At 3-month follow-up, 2.30 ± 2.18 RPD were classified as Stage 1, 8.75 ± 4.79 as Stage 2, 1.15 ± 2.56 as Stage 3, and 0.20 ± 0.70 as Stage 4 (Fig. 3). This accounted for a significant increase of Stage 1 RPD during the follow-up (*p* = 0.002), no significant changes of Stage 2 RPD (*p* = 0.909), but a significant decrease of Stage 3 RPD (*p* = 0.020) and no significant changes of Stage 4 RPD (*p* = 0.630). This improvement was mainly due to a general improvement in stage 2 and 3 RPD. In detail, 62% of stage 3 RPD (41 out of 66 stage 3 RPD of all patients) showed an improvement during the 3-month follow-up (28 out of 66 RPD from stage 3 to stage 2, and 13 out of 66 RPD from stage 3 to stage 1). Furthermore, 16% of stage 2 RPD (27 out of 172 of stage 2 RPD of all patients) showed an improvement to stage 1 during the 3-month follow-up.

Another area of 1.27 mm^2^ with a similar distance from the fovea (1901 ± 528 µm, *p* = 0.689) was selected as a control area (i.e. area without treatment). In the control area, we identified a mean of 9.6 ± 4.6 RPD (median 9.5, range 4–20) (*p* = 0.127). Analyzing the distribution of RPD among the RPD stages in the control area (i.e. area without treatment), no significant changes were observed during the follow-up in different stages of RPD. In detail, at the baseline, 0.56 ± 0.24 RPD were classified as Stage 1 (0.50 ± 1.04 and 0.22 ± 0.55 RPD at 1-month and 3-month follow-up, respectively; *p* = 0.157). At the baseline, 5.61 ± 3.15 RPD were classified as Stage 2 (5.67 ± 3.61 and 6.06 ± 3.56 RPD at 1-month and 3-month follow-up, respectively; *p* = 0.305), 3.83 ± 3.75 RPD were classified as Stage 3 (3.28 ± 3.89 and 3.22 ± 3.73 RPD at 1-month and 3-month follow-up, respectively; *p* = 0.251), and 0.11 ± 0.32 RPD were classified as Stage 4 (0.17 ± 0.51 and 0.11 ± 0.32 RPD at 1-month and 3-month follow-up, respectively; *p* = 0.331).

**Figure 4:**
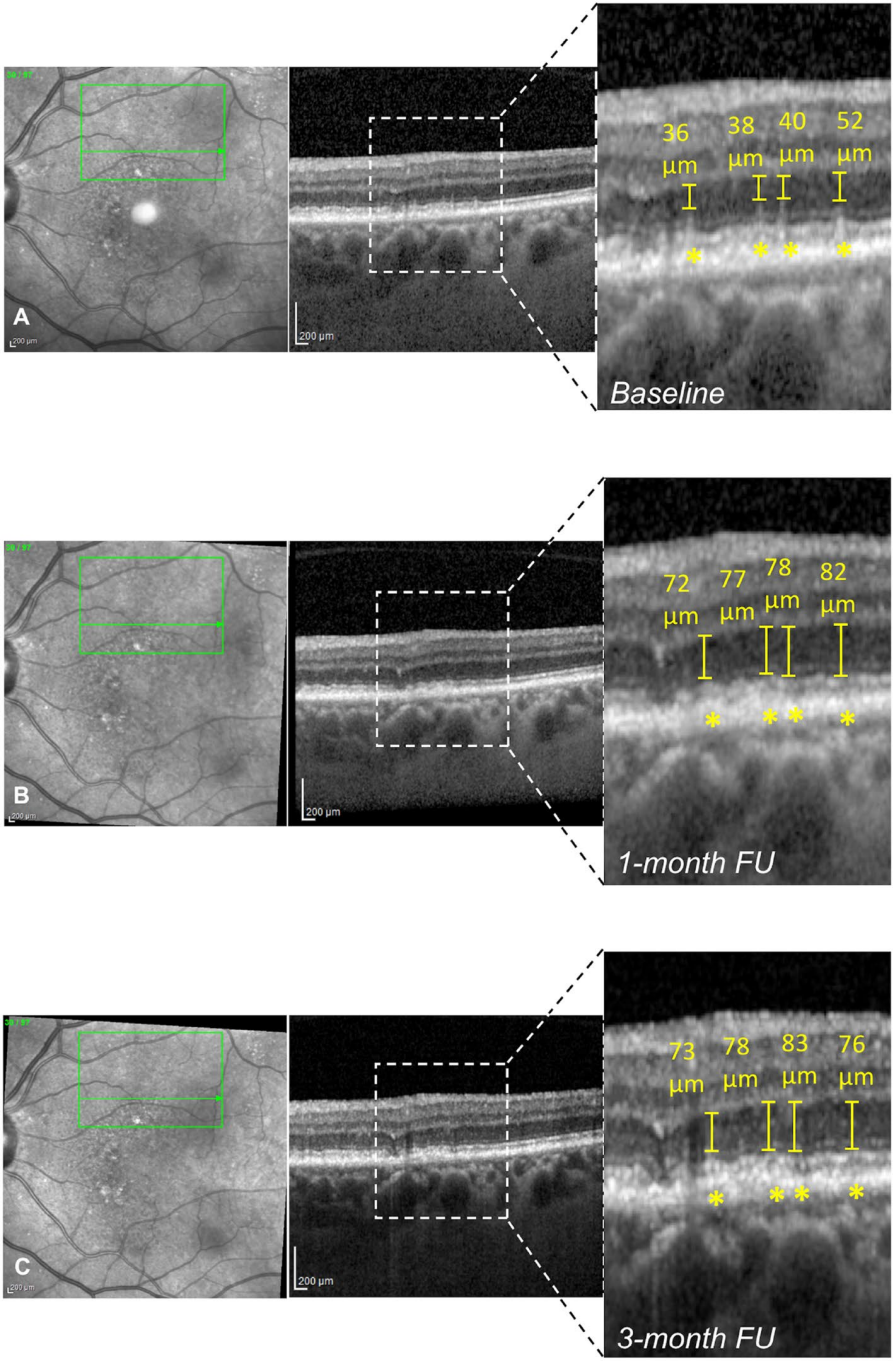
Structural optical coherence tomography (OCT) changes in the morphology of the outer nuclear layer (ONL) before and after subthreshold laser treatment. (**A**) Combined infrared reflectance (IR) and structural OCT showing the presence of 4 reticular pseudodrusen (RPD) in the treated area at the baseline (magnification). (**B**, **C**) Combined IR and structural OCT showing the partial regression of RPD with increased ONL thickness at 1-month (**B**) and 3-month follow-up (**C**) (magnifications).

Analyzing the treated area, the thickness of the ONL significantly increased during the follow-up (*p* = 0.001). In detail, mean ONL thickness at the baseline was 59.30 ± 13.50 µm (median 60.5, range 32–84) and increased to 64.75 ± 12.31 µm (median 69, range 41–82) at 1-month follow-up (*p* = 0.012) and to 67.75 ± 15.52 µm (median 71, range 36–93) at 3-month follow-up (*p* < 0.001) (Table 2, Fig. 4). On the other hand, analyzing the control area, the ONL thickness was 51.61 ± 10.91 µm at the baseline (median 55.5, range 22–69) and it did not show any significant change during the follow-up (53.39 ± 12.29 µm, median 54, range 23–65 [*p* = 0.645], and 53.56 ± 12.31 µm, median 58, range 17–66 [*p* = 0.204] at 1-month and 3-month follow-up, respectively).

Interobserver variability between readers was excellent for all measurements (ICC = 0.965 [0.912–0.986]).

No significant changes during the follow-up were observed analyzing the mean retinal thickness and choroidal thickness in the treated area (*p* = 0.238 and *p* = 0.450, respectively) (Table 2).

Analyzing FAF images, no development/extension of atrophic lesions was observed during the follow-up. Furthermore, no changes in the appearance of RPD were observed.

### Safety analysis

No topical and/or systemic side effects were reported from the patients during the 3-month follow-up. None of the patients developed a MNV in the treated eye, and no eye developed atrophic lesion during the follow-up detected using FAF. No other retinal changes in the treated area were disclosed using multimodal imaging modality.

## Discussion

Here, we have reported the results of the first pilot study investigating the safety and short-term efficacy of SLT in patients affected by RPD secondary to dAMD. Overall, the results of our single-center, interventional clinical trial have confirmed the safety of end-point management yellow SLT in the treatment of RPD. Indeed, the MS in both macular and treated areas did not show worsening during the 3-month follow-up using the microperimetry. Furthermore, no ocular or systemic side effects were reported in our patients.

“High-density/low-intensity” SLT was first reported in 2005 in the treatment of diabetic macular edema (DME)^27^. Subthreshold laser does not cause retinal damage and has no known adverse treatment effects^21,28,29^. Indeed, it has been demonstrated that both subthreshold infrared laser and subthreshold yellow laser do not cause clinically visible or invisible scars in the macula^29,30^, and that SLT can be used transfoveally in eyes with 20/20 visual acuity to reduce the risk of visual loss caused by early fovea-involving DME^24^. Our data have confirmed that SLT is safe also in patients with high BCVA and dAMD. No local and systemic adverse events were detected after 3 months from the laser treatment (i.e. no slit lamp or fundus abnormal findings, no crystalline lens changes in phakic eyes, no IOP changes, no development of MNV in the treated area).

The PASCAL clinical trial, is a single-center pilot study, evaluating the effect of end-point management yellow SLT in patients with RPD secondary to dAMD. Reticular pseudodrusen deposits are characterized by a widespread disruption and loss of RPE and ellipsoid zone inducing an impaired retinal sensitivity and worse visual function already from the early stages of the disease^14^. Furthermore, patients with RPD are characterized by a faster progression to both forms of late AMD^14,18,19^. It has been suggested that subthreshold laser works by targeting, preserving, and “normalizing” the function of the RPE^31,32^. Luttrull et al^23^ have suggested a wider role for subthreshold laser as retinal reparative/protective therapy by re-establishing the function of RPE in patients affected by nAMD, restoring the drug response in drug-tolerant eyes. As subthreshold laser seems to play a role in restoring the function of RPE in patients affected by AMD^23^ and dysfunction of the RPE has been suggested as the main driving factor in the pathogenesis of RPD, SLT could play a crucial role in the regression of RPD. Our pilot study showed interesting results in the anatomical outcomes of patients treated with subthreshold laser. In detail, analyzing the stages of RPD in the treated area at the baseline and at follow-up examinations, we reported a significant decrease in the number of stage 3 RPD (*p* = 0.020) with, simultaneously, a stage 1 RPD increase at 3-month follow-up after the laser treatment (*p* = 0.002). Furthermore, according to the down-staging of RPD, we observed a significant increase in the ONL thickness above the treated RPD during the FU (*p* = 0.001). On the other hand, no changes in RPD distribution and no changes in ONL thickness were observed in the control area (i.e. area without treatment). These data suggest a real regression of the RPD rather than a RPD resorption due to the progression of the disease. Indeed, Spaide et al^33^ reported outer retinal atrophy (i.e. decrease of ONL thickness) in patients with regression of the RPD due to the natural history and evolution of the disease. Contrary, our series demonstrated an increase of the ONL after the reabsorption of RPD secondary to the SLT, supporting the theory of the regression of the disease. These results are of fundamental importance because a regression of the stages of RPD due to SLT could reduce the risk of developing an advanced form of AMD, both neovascular or atrophic. One can argue that our study did not show any significant functional improvement in terms of BCVA or MS in both foveal and treated areas after the treatment. However, we need to account for different aspects. First of all, we treated a small area outside the central fovea, and, for this reason, BCVA may not show any significant change. Secondly, no improvement of MS could be explained by the small treated area and by the short-term follow-up of our clinical trial. On the other side, being a pilot study, a short follow-up was necessary to early detect the possible adverse events. Further studies, with a longer follow-up and a spread treatment involving a larger area, should be performed in order to confirm the results of our pilot study.

Recently, a randomized controlled clinical trial, the LEAD study^34^, demonstrated that subthreshold nanosecond laser (SNL) is a safe treatment but did not significantly reduce the overall rate of the progression to late AMD (both GA and nAMD) in patients with intermediate AMD. However, a post-hoc analysis showed a possible benefit of SNL in patients without RPD and a worse outcome in patients with RPD^34,35^. These data could seem in contrast with our series. However, several differences should be kept in mind. First of all, in the LEAD study, the effect of SNL on patients with RPD was investigated in a post-hoc analysis and not as pre-specified outcome of the study; thus, these data should be taken with caution. Importantly, differently from our study, no detailed morphological and functional analysis of treated RPD was carried out in the LEAD study preventing any definite role of subthreshold laser in the treatment of RPD. Finally, our study is not comparable with the LEAD study because two different kinds of laser were used: the LEAD study used a subthreshold nanopulsed laser, whereas we used a subthreshold continuous laser with end-point management. The different effects on RPD could be due to the different mechanisms of action of the two lasers on the RPE cells.

Despite the encouraging results of the PASCAL clinical trial, it is necessary to clarify the limits of our study, as the low number of patients included in our sample, the short term follow-up, and the margin of error of the retinal Heidelberg tracking system, which may have slightly distorted the lecture of OCT dense scans at the follow-up. However, this is a pilot study aimed to test the safety of the SLT in patients with RPD secondary to dAMD, providing encouraging results and thus suggesting a potential benefit for such approach.

In conclusion, we report the short-term safety of the end-point management yellow SLT with Pascal Synthesis 577 nm in the treatment of RPD. Furthermore, our results suggest that it may be effective in inducing RPD regression in terms of RPD stage and ONL thickening. However, further clinical studies with larger sample sizes, a sham group, and a longer follow-up are needed in order to confirm these promising results and to test the effects on the progression rate to late AMD of the treated patients.

## Supplementary Information

Supplementary Information 1.

Supplementary Information 2.
