# Supplementary material for: Subthreshold laser treatment for reticular pseudodrusen secondary to age-related macular degeneration
Source: Sci Rep. 2021 Jan 26;11:2193. doi: 10.1038/s41598-021-81810-7 (PMC7838261; doi:10.1038/s41598-021-81810-7)
Supplement: Supplementary file 1 — Supplementary Information 1. [file 41598_2021_81810_MOESM1_ESM.docx]

**-Original Article-**

**Subthreshold laser treatment for reticular pseudodrusen secondary to age-related macular degeneration**

Giuseppe Querques, MD, PhD (1)(2)*, Riccardo Sacconi, MD, FEBO (1)(2)*, Francesco Gelormini, MD (1)(2), Enrico Borrelli, MD, FEBO (1)(2), Francesco Prascina, MD, (2), Ilaria Zucchiatti, MD (2), Lea Querques, MD (2), Francesco Bandello, MD, FEBO (1)(2)

(1) School of Medicine, Vita-Salute San Raffaele University, Milan, Italy

(2) Division of head and neck, Ophthalmology Unit, IRCCS San Raffaele Scientific Institute, Milan, Italy

*These authors contributed equally to this study and should be considered equivalent authors

**Inclusion and exclusion criteria of the PASCAL clinical trial**

Inclusion Criteria:

- 50 years or older;
- Diagnosis of dry Age-related Macular Degeneration (AMD);
- Presence of reticular pseudodrusen (RPD);
- Best corrected visual acuity between 20/20 and 20/400 inclusive;
- Clear ocular media;
- Ability to provide informed consent and attend all study visits.

Exclusion Criteria:

- Evidence of late AMD;
- Presence of choroidal neovascularization in the included eye;
- Any prior treatment for AMD, aside from antioxidants in the included eye;
- Any other ocular condition that would progress in the study period and confound visual acuity assessment;
- Any ocular or systemic medication known to be toxic to the lens, retina or optic nerve;
- Presence of idiopathic or autoimmune-associated uveitis;
- Any intraocular surgery 3 months of entry;
- Any prior thermal laser in the macula;
- History of vitrectomy, filtering surgery, corneal transplant or retinal detachment surgery;
- Previous therapeutic radiation in the ocular region in either eye;
- Any treatment with an investigational agent in the previous 60 days before study entry;
- Women of child-bearing potential, defined as all women less than 1 year postmenopausal or less than 6 weeks since sterilization at Baseline, unless they are using highly effective methods of contraception during dosing of study treatment;
- Participation in an investigational drug, biologic, or device study within 6 Months prior to Baseline.
