# Supplementary material for: Subthreshold laser treatment for reticular pseudodrusen secondary to age-related macular degeneration
Source: Sci Rep. 2021 Jan 26;11:2193. doi: 10.1038/s41598-021-81810-7 (PMC7838261; doi:10.1038/s41598-021-81810-7)
Supplement: Supplementary file 2 — Supplementary Information 2. [file 41598_2021_81810_MOESM2_ESM.docx]

**-Original Article-**

**Subthreshold laser treatment for reticular pseudodrusen secondary to age-related macular degeneration**

Giuseppe Querques, MD, PhD (1)(2)*, Riccardo Sacconi, MD, FEBO (1)(2)*, Francesco Gelormini, MD (1)(2), Enrico Borrelli, MD, FEBO (1)(2), Francesco Prascina, MD, (2), Ilaria Zucchiatti, MD (2), Lea Querques, MD (2), Francesco Bandello, MD, FEBO (1)(2)

(1) School of Medicine, Vita-Salute San Raffaele University, Milan, Italy

(2) Division of head and neck, Ophthalmology Unit, IRCCS San Raffaele Scientific Institute, Milan, Italy

*These authors contributed equally to this study and should be considered equivalent authors

# PROTOCOL SUMMARY

| **Title** | Subthreshold laser treatment for reticular pseudodrusen secondary to age-related macular degeneration: the PASCAL clinical trial |
| --- | --- |
| **Objectives** | To evaluate the effectiveness of subthreshold laser treatment on retinal sensitivity in patients with reticular pseudodrusen secondary to AMD. Secondary objective is to investigate changes in best-corrected visual acuity, atrophy progression and safety |
| **Study Design** | Pilot, single-center, interventional clinical trial in which subjects will receive subthreshold laser treatment on a customized extramacular area of 1,27 mm^2^ |
| **Study Population** | Patients with reticular pseudodrusen. |
| **Number of Subjects** | 20 subjects |
| **Gender/Age** | Males and females, 50 years of age or older. |
| **Number of Centers** | Single site. |
| **Key Eligibility Criteria** | Inclusion Criteria:   - Reticular pseudodrusen - 50 years or older - The periphery of the atrophic lesions must demonstrate increased autofluorescence - Best corrected visual acuity between 20/20 and 20/400 inclusive - Clear ocular media - Ability to provide informed consent and attend all study visits   Exclusion Criteria:   - GA secondary to other causes aside from AMD - Any prior treatment for AMD, aside from antioxidants - Any corneal opacity, cataract formation and hemorrhage in the vitreous body, which may interfere with viewing by the laser surgeon of the target structures - Aphakic eye with vitreous in the anterior chamber - Neovascular Glaucoma - Glaucoma caused by congenital angle anomalies - Open angle of less than 90º or extensive peripheral anterior and low synechia, present circumferentially around the corner - Significant corneal edema or reduced water clarity that obscures the view angle in detail - Glaucoma secondary to active uveitis - Any other ocular condition that would progress in the study period and confound visual acuity assessment - Any ocular or systemic medication known to be toxic to the lens, retina or optic nerve Presence of idiopathic or autoimmune-associated uveitis - Any intraocular surgery 3 months of entry - Any prior thermal laser in the macula - History of vitrectomy, filtering surgery, corneal transplant or retinal detachment surgery - Previous therapeutic radiation in the ocular region in either eye - Any treatment with an investigational agent in the previous 60 days before study entry - Women of child-bearing potential, defined as all women less than 1 year postmenopausal or less than 6 weeks since sterilization (further definition can be found in Section 12.7) at Baseline, unless they are using highly effective methods of contraception during dosing of study treatment. - Participation in an investigational drug, biologic, or device study within 6 Months prior to Baseline [Note: observational clinical studies solely involving over-the-counter vitamins, supplements, or diets are not exclusionary |
| **Investigational Product** | The Pascal Synthesis 577 is Topcon’s top-of-the-line laser photocoagulator combining advanced PASCAl scanning technology with the clinical benefits of the 577 nm yellow wavelength.  Wavelength: 577 nm  Power: 0-2000mW |
| Study Procedures | Subjects will be screened for inclusion into the study after providing written informed consent. Screening visit includes best-corrected visual acuity, ophthalmic examination, intraocular pressure, Spectral Domain - Optical Coherence Tomography (SD-OCT) and blue auto-fluorescence (BAF).  On Day 0 subjects will undergo best-corrected visual acuity, ophthalmic examination, intraocular pressure, Spectral Domain - Optical Coherence Tomography (SD-OCT), BAF and customized microperimetry evaluation on a extramacular rectangular area (1.27 mm^2^) later treated with subthreshold laser. The patient will return for follow-up 4 weeks and 12 weeks after laser treatment. During all these follow-up visits the patient will undergo complete ophthalmic evaluation, included SD-OCT, BAF and microperimetry on the treated area.  Screening visit and Day 0 can be performed on the same day. |
| Clinical Parameters | The following clinical parameters will be evaluated:   1. Slit lamp and fundus examinations 2. Intraocular pressure 3. Best corrected visual acuity (ETDRS) 4. BAF 5. Optical coherence tomography and autofluorescence 6. Microperimetry 7. Adverse events |
| **Efficacy Endpoints** | Primary efficacy measure:  Change in retinal sensitivity on customized microperimetry at 12 weeks.  Other efficacy measures:  Change in mean VA  Change in structural OCT |
| **Safety Endpoints** | Ocular safety will be evaluated by assessing:  Adverse Events (AEs)  Serious adverse events (SAEs)  Moderate or significant visual acuity loss  Slit lamp findings  Fundus findings  Crystalline lens changes in phakic eyes  Intraocular pressure |

1. **BACKGROUND**

## Age-Related Macular Degeneration Overview

Age-related macular degeneration (AMD) is the most common cause of visual impairment and blindness in the elderly in industrialized nations.^1^ AMD is a degenerative disorder affecting the macula and it is classified into two main forms: non-neovascular (also known as “dry” or “nonexudative”) or neovascular (also known as “wet” or “exudative”). Dry AMD is the most common form, comprising around 90% of diagnosed disease.^2^ Geographic atrophy (GA) is the advanced stage of dry AMD, where areas of atrophy become confluent and cause visual loss. Wet AMD, characterized by choroidal neovascularization (CNV) - abnormal blood vessels typically arising in the choriocapillaris and often invading the subretinal space - is associated with rapid progression to advanced sight loss.^2^

Early AMD is characterized by the presence of specific clinical findings including drusen, reticular psuedodrusen and RPE changes. Drusen are yellowish deposits at the level of the RPE which lies just under the neurosensory retina.^4^ Pseudodrusen are hyperreflective material located not under (as typical drusen of AMD do) but above the RPE and are a high risk factor in the progression of AMD (both CNV and GA).^5^

In particular, several studies have highlighted the role of reticular pseudodrusen to accelerate the rate of GA progression.^5^

GA is characterized by the degeneration of the retinal pigment epithelium (RPE), photoreceptor cells and choriocapillaris.^6^ These morphological changes initially appear in the extrafoveal region and advance into the fovea as the disease progresses. In earlier stages when the disease is limited to the extrafoveal region, the atrophic lesions impair visual performance by limiting the size of the functioning fovea and patients may be able to see only a portion of words when reading. As the disease progresses to include the parafoveal region, central vision becomes affected. Severe vision loss occurs when these areas of atrophy enlarge or coalesce and expand into the foveola. In addition to the formation of scotomas that progress into the foveola, patients with GA can experience a variety of visual symptoms such as distorted vision, trouble discerning colors, a slow recovery of visual function after exposure to bright light, a loss of contrast sensitivity and marked visual impairment in dimly lit environments.^7,8,9^

## Historical and Current Treatment of AMD

At present, proven therapies to prevent or treat AMD are the following: (i) antioxidant vitamins and minerals for slowing the progression to later stages of AMD; (ii) intravitreal injection of anti-VEGF agents, (iii) photodynamic therapy (PDT), and (iv) laser photocoagulation to treat neovascular AMD. These data are supported by several prospective randomized controlled clinical trials^10^.

Antioxidant vitamins and minerals (as suggested in the original AREDS and AREDS2 reports) are recommended for those patients with (i) intermediate AMD and (ii) advanced AMD in one eye.

Intravitreal injections of anti-VEGF agents have become first-line therapy for treating and stabilizing most cases of neovascular AMD ^11,12,13,14^, since the introduction of the VEGF inhibitors (i) pegaptanib sodium (Macugen®, Eyetech, Inc., Cedar Knolls, NJ) in 2004, off-label (ii) bevacizumab (Avastin®, Genentech, Inc., South San Francisco, CA) in 2005, (iii) ranibizumab (Lucentis®, Genentech, Inc., South San Francisco, CA) in 2006, and (iv) aflibercept (Eylea™, Regeneron Pharmaceuticals, Inc., Tarrytown, NY) in 2011.

Unlike the other anti-VEGF agents that are currently available (ranibizumab, aflibercept, and bevacizumab), pegaptanib treatment does not improve visual acuity on average in patients with new-onset neovascular AMD and is rarely used in current clinical practice.

Drug tolerance is the most common cause of treatment failure in neovascular age-related macular degeneration. It has been demonstrated that “low-intensity/high-density” subthreshold diode micropulse laser (SDM) treatment restores responsiveness to anti–VEGF agents in drug-tolerant eyes by targeting, preserving, and normalizing the function of the RPE ^15^.

PDT with verteporfirin (Visudyne®) is recommended for selected type of neovascular AMD ^16,17^.

Thermal laser photocoagulation surgery may be considered for extrafoveal CNV, new or recurrent ^18^.

However, there are no effective treatments for early AMD to prevent the progression to GA.

In particular, there are currently no treatments for reticular pseudodrusen, which are considered as high-risk lesions for progression to GA. Moreover, there is no proven therapy to prevent or treat GA that occurs in dry AMD. In particular, there are currently no treatments for earlier stages, when the disease is limited to the extrafoveal region (extramacular GA <1.27 mm^2^ area), to prevent the progression of atrophy into the fovea.

## Background and rationale on subthreshold laser treatment

“High-density/low-intensity” subthreshold laser treatment was first reported in 2003.^19^ By definition, subthreshold laser does not cause retinal damage and has no known adverse treatment effect.^20-23^ Subthreshold laser selectively targets the RPE, and has been reported to be an effective treatment in a number of retinal disorders, including diabetic macular edema, proliferative diabetic retinopathy, macular edema as a result of branch retinal vein occlusion, and central serous chorioretinopathy.^20-23^ It has been suggested that subthreshold laser works by targeting, preserving, and “normalizing” (moving toward normal) the function of the RPE. A recent publication of Luttrull et al.^24^ demonstrated that subthreshold laser treatment restored drug response in drug-tolerant eyes with neovascular AMD. Subthreshold laser does this without inducing any morphologic change in the RPE or causing even transient break-down in the blood–retinal barrier. The paper of Luttrull et al.^24^ suggests a wider role for subthreshold laser as retinal reparative/protective therapy by re-establishing the function of RPE.

Our purpose is to evaluate the effect of subthreshold laser treatment in patients with reticular pseudodrusen and incipient GA. Reticular pseudodrusen deposits are characterized a widespread disruption and loss of RPE and ellipsoid (previously known as IS/OS interface) inducing an impaired retinal sensitivity, significantly reduced in eyes with reticular pseudodrusen compared with eyes with typical drusen despite good visual acuity in both groups. Dysfunction of the RPE has been suggested as the main driving factor in the pathogenesis of reticular pseudodrusen. Supported by the study of Luttrull et al.^24^ we believe that subthreshold laser should restore the RPE function in eyes with pseudodrusen and incipient GA offering a reparative/protective effect.

## Clinical data

“High-density/low-intensity” subthreshold laser (SDM) treatment was first reported in 2005^25^. By definition, SDM does not cause retinal damage and has no known adverse treatment effect. Subthreshold laser has been reported to be an effective treatment in a number of retinal disorders, including diabetic macular edema (DME), proliferative diabetic retinopathy (DR), macular edema as a result of branch retinal vein occlusion, and central serous chorioretinopathy^26^.

Moreover, Luttrull JK et al restored drug response by using SDM treatment in consecutive eyes unresponsive to all anti–vascular endothelial growth factor drugs^24^.

The safety of SDM is such that it may be used transfoveally in eyes with 20/20 visual acuity to reduce the risk of visual loss caused by early fovea-involving DME^26^. Furthermore it has been demonstrated that both subthreshold infrared laser (IR-MPL) and subthreshold yellow laser (Y-MPL) do not cause clinically visible or invisible scars in the macula and that they seem to be safe from the morphologic and visual function points of view in mild center-involving diabetic macular edema^27^.

Finally, Kenichiro B, et al demonstated that immediately after subthreshold laser treatment, autofluorescence imaging was more sensitive to detect RPE changes than fluorescein angiography, suggesting that noninvasive autofluorescence imaging may allow prediction of the effect of subthreshold laser treatment and might be used to titrate treatment dose^28^.

Finally, it has been observed that laser photocoagulation of drusen leads to their disappearance. However, treatment does not result in a reduction in the risk of developing CNV, and was not shown to limit the occurrence of geographic atrophy or visual acuity loss. Ongoing studies are being conducted to assess whether the use of extremely short laser pulses (i.e. nanosecond laser treatment) cannot only lead to drusen regression but also prevent neovascular AMD.29

# STUDY DESIGN

## Objective

The objective of this study is to establish the safety of subthreshold laser treatment in patients with reticular pseudodrusen.

## Description of the study

Approximately 20 naïve patients with reticular pseudodrusen who underwent subthreshold laser treatment in a 1.27 mm^2^ area.

Subjects will be evaluated at Screening/Baseline and then 12 weeks and 24 weeks after laser treatment with a full ocular examination, visual acuity measurement (VA), optical coherence tomography (OCT) with autofluorescence and microperimetry.

## Rationale for Study Design

The rationale of the study is to prevent the evolution of reticular pseudodrusen to Atrophic degeneration.^5,7,8,9^

## Outcome Measures

**Primary efficacy measure at week 12:**

Change in retinal sensitivity on customized microperimetry.

**Other efficacy measures at week 12:**

Change in mean VA

Change in structural OCT findings in the treated area

**Safety Outcome Measures**

Incidence of adverse events (AEs)

Serious adverse events (SAEs)

Moderate or significant visual acuity loss

Slit lamp findings

Crystalline lens changes in phakic eyes

Intraocular pressure

Fundus findings

## Study Population

Up to 20 subjects with reticular pseudodrusen will be enrolled. After providing informed consent and documenting it in writing, subjects will be screened for participation in the study. Screening evaluations may be performed at any time within the 14 days preceding and subjects must fulfill the following criteria:

### Inclusion Criteria

- Reticular pseudodrusen
- 50 years or older
- The periphery of the atrophic lesions must demonstrate increased autofluorescence
- Best corrected visual acuity between 20/20 and 20/400 inclusive
- Clear ocular media
- Ability to provide informed consent and attend all study visits

### Exclusion Criteria

- GA secondary to other causes aside from AMD
- Any prior treatment for AMD, aside from antioxidants
- Any corneal opacity, cataract formation and hemorrhage in the vitreous body, which may interfere with viewing by the laser surgeon of the target structures
- Aphakic eye with vitreous in the anterior chamber
- Neovascular Glaucoma
- Glaucoma caused by congenital angle anomalies
- Open angle of less than 90º or extensive peripheral anterior and low synechia, present circumferentially around the corner
- Significant corneal edema or reduced water clarity that obscures the view angle in detail
- Glaucoma secondary to active uveitis
- Any other ocular condition that would progress in the study period and confound visual acuity assessment
- Any ocular or systemic medication known to be toxic to the lens, retina or optic nerve
- Presence of idiopathic or autoimmune-associated uveitis
- Any intraocular surgery 3 months of entry
- Any prior thermal laser in the macula
- History of vitrectomy, filtering surgery, corneal transplant or retinal detachment surgery
- Previous therapeutic radiation in the ocular region in either eye
- Any treatment with an investigational agent in the previous 60 days before study entry
- Women of child-bearing potential, defined as all women less than 1 year postmenopausal or less than 6 weeks since sterilization (further definition can be found in Section 12.7) at Baseline, unless they are using highly effective methods of contraception during dosing of study treatment.Effective contraception methods include:
- Total abstinence (when this is in line with the preferred and usual lifestyle of the subject). Periodic abstinence (eg, calendar, ovulation, symptothermal, postovulation methods) and withdrawal are not acceptable methods of contraception
- Female sterilization (have had surgical bilateral oophorectomy with or without hysterectomy) or tubal ligation at least 6 weeks before Baseline. In case of oophorectomy alone, only when the reproductive status of the woman has been confirmed by follow up hormone level assessment
- Male sterilization (at least 6 months prior to Baseline). For female subjects in the study, the vasectomized male partner should be the sole partner for that subject
- Use of oral, injected or implanted hormonal methods of contraception or other forms of hormonal contraception that have comparable efficacy (failure rate < 1%), for example hormone vaginal ring or transdermal hormone contraception
- Placement of an intrauterine device (IUD) or intrauterine system (IUS)
- Participation in an investigational drug, biologic, or device study within 6 Months prior to Baseline [Note: observational clinical studies solely involving over-the-counter vitamins, supplements, or diets are not exclusionary

In cases where both eyes are eligible, the eye with the worse BCVA at Baseline will be selected as the study eye. If both eyes have the same BCVA, it is recommended to select the right eye as the study eye.

### Withdrawal Criteria

Subjects have the right to withdraw from the study at any time, for any reason, without

jeopardizing their medical care. Where possible, subjects will be followed for safety and

encouraged to return for follow-up visits for any unresolved safety events.

Subjects who withdraw from the study for any reason at any time will not be replaced.

Subjects who voluntarily withdraw from the study prior to the Week 12 Visit (EOS) will be

asked to complete all procedures outlined in the Week 12 visit.

## Compliance with Standards

This study will be conducted in accordance with applicable state, national, and international standards. The Istitution will have attained the appropriate state and local licensing requirements permitting the utilization of Pascal Synthesis 577 system. Personnel will be licensed to operate laser equipment as required by applicable state regulations.

The Pascal Synthesis 577 system will be delivered by Topcon with an operating manual. During the study, Topcon personnel will be available as needed to support the investigators. In addition, investigators will undergo training on Pascal Synthesis 577 system operation prior to study initiation.

# STUDY TREATMENT

## Subthreshold laser treatment

PASCAL Streamline 577™ combines the optimum 577 nm wavelength with the most complete and comprehensive scanning laser configuration. The Streamline 577 offers ophthalmologists a far superior treatment solution.

The longer wavelength of the 577 better targets the RPE with less scatter than 532 or 561 nm lasers. The combined absorption by both melanin and the oxyhemoglobin makes the 577 more efficient. Energy is concentrated to a smaller volume allowing use of lower powers and shorter pulse durations.


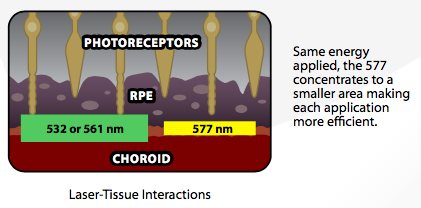


What is Subthreshold?

Subthreshold photocoagulation is a method where the burn spots cannot be seen with biomicroscopy. With subthreshold photocoagulation, the treated areas cannot be seen on color fundus photograph, but they also cannot be seen on OCT or FA when the subthreshold level is adequately set. Subthreshold photocoagulation can be considered to be truly minimally invasive or non-damaging photocoagulation. A term sometimes used is photo-thermal stimulation since the RPE layer is heated and stimulated, but not destroyed.

The threshold level output power is set to obtain barely visible burn at approximately 200mW to 250mW using the titration mode, and irradiation is conducted after switching over to Endpoint Management.

# STUDY PROCEDURES

## Informed Consent

Informed consent must be obtained and documented in writing prior to the initiation of any study procedures. The subject (or the subject's legally authorized representative) must be allowed sufficient time to thoroughly read (or have explained), the informed consent form, which will be written at a level that can be understood by someone educated to an 8^th^ grade level. The Investigator or his/her designee should answer any questions that the subject/representative might have. If the subject agrees to participate in the study, i.e., provides informed consent, the subject/representative must sign both copies of the informed consent form. The witness and the Investigator must also sign both copies of the informed consent form. One copy of the informed consent form should be given to the subject/representative. If applicable, it will be provided in a certified translation of the local language. The date of the subject’s signature on the informed consent form should be noted in the subject’s medical chart.

Subjects who provide written informed consent will be screened for eligibility and once they are determined as being eligible, they will be enrolled into the study.

## Assignment of Subject Identification

A subject identification number (ID) will be assigned at screening. This ID should be used on all study-related documents. To maintain confidentiality, the subject’s name should not be recorded on any study document other than the informed consent form.

## Screen Failure

A record of screen failures and the reasons for the screen failures will be maintained by the investigator.

## Concomitant and Excluded Therapies

Concomitant medications are any prescription drugs or devices used by a subject during the study. The CRF will record administration of these medications.

No other treatments for GA and/or reticular is permitted for the study eye during the trial, except the use of alimentary supplements.

Investigators are reminded to avoid enrolling any subject likely to require cataract surgery during the 12 weeks study period.

No other experimental or investigational treatments are allowed during this study, including ocular experimental and investigational treatments in either eye.

## Timing of Study Assessments

### Screening (Day -14 to Day 0)

After obtaining informed consent, the following study assessments will be performed within 14 days prior to Day 0 and all ocular assessments will be performed**:**

- Demographic information
- Medical history and record of concomitant medications
- Ophthalmic history, including prior medication use
- Best Corrected ETDRS Visual Acuity (VA) at 4 meters prior to dilatation
- Slit lamp examination
- Fundus examination (by indirect ophthalmoscopy)
- Intraocular pressure (IOP)
- Spectral Domain Optical Coherence Tomography (SD-OCT)
- BAF in the area later treated with laser (Autofluorescence)
- Microperimetry in a customized area later treated with laser

### Day 0 (Baseline) – Treatment Visit

- Ophthalmic history, including prior medication use
- Best Corrected ETDRS Visual Acuity (VA) at 4 meters prior to dilatation
- Slit lamp examination
- Fundus examination (by indirect ophthalmoscopy)
- Intraocular pressure (IOP)
- Spectral Domain Optical Coherence Tomography (SD-OCT)
- BAF in the area later treated with laser (Autofluorescence)
- Microperimetry in a customized area later treated with laser
- An extramacular area of 1.27 mm2 ( ½ of a disk area, disk area= 2.54 mm^2^ ) is treated with subthreshold laser.
- Record concomitant medications
- Post treatment assessment

Screening visit and Day 0 can be performed on the same day

### Follow-up Visits (week 4 +/- 7 days , week 12 +/- 7 days)

- BVCA
- Slit lamp examination
- Fundus examination (by indirect ophthalmoscopy)
- IOP
- Spectral Domain Optical Coherence Tomography (SD-OCT)
- BAF in the area previously treated with laser (Autofluorescence)
- Microperimetry in a customized area previously treated with laser
- Record concomitant medications

### Unscheduled Visits

If at any time during the study, outside of the above scheduled visits, the physician determines the subject should be assessed for re-treatment, an unscheduled visit may occur and the following assessments should be performed on **the study eye**:

- Best Corrected ETDRS Visual Acuity (VA) at 4 meters prior to dilatation
- Slit lamp examination
- Fundus examination (by indirect ophthalmoscopy)
- Intraocular pressure (IOP)
- Spectral Domain Optical Coherence Tomography (SD-OCT)
- BAF in a customized area treated by laser (Autofluorescence)
- Microperimetry in a customized area treated by laser
- Record adverse events
- Record concomitant medications

### Follow Up of Discontinued Subjects

Subjects who discontinue the study will be followed for safety where possible and encouraged to return for follow-up visits for any unresolved safety events.

Subjects who voluntarily withdraw from the study will be asked to complete procedures outlined in the Week 12 visit. Subjects who are withdrawn due to adverse events will be followed at least until resolution or stabilization of the adverse event.

All procedures should be completed as soon as possible after the determination is made to discontinue the subject from the study. If the subject will remain in the study for safety evaluation, follow-up visits should be scheduled according to the schedule of visits and procedures.

# EFFICACY PARAMETERS

Efficacy assessments will include best corrected visual acuity (BCVA), optical coherence tomography (OCT), BAF and microperimetry. Whenever possible, the same person should perform the evaluations specified by the protocol at each study visit. Unless otherwise indicated, all ocular assessments should be performed on the study eye only.

## ETDRS Best-Corrected Visual Acuity (VA)

Manifest refraction and VA measurement must be performed according to the standard procedure originally developed for ETDRS.

## Blue auto-fluorescence

BAF will be used to analyze atrophy as a well-defined hypo-autofluorescence area.

## Spectral Domain-Optical Coherence Tomography (SD-OCT)

SD-OCT will be used to analyze the outer retinal morphology in the treated area

## Microperimetry

Microperimetry will be used to asses improving of sensibility in the customized treated area.

# SAFETY PARAMETERS

Safety assessments include incidence of adverse events/serious adverse events, visual acuity, slit lamp findings, crystalline lens changes in phakic eyes, intraocular pressure, and fundus findings. The reporting time period is from day 0 through the last study visit (Week 12).

## Definitions of Adverse Events

### Adverse Events (AE)

An AE is any unfavorable and unintended sign, symptom, or disease temporally associated with the use of an investigational device or other protocol-imposed intervention, regardless of attribution. This includes the following:

- Any untoward medical occurrence not previously observed in the subject that emerges during the protocol-specified AE reporting period, including signs or symptoms associated with AMD that were not present prior to the AE reporting period.
- Complications that occur as a result of protocol-mandated interventions (e.g., invasive procedures such as injection procedures).
- Preexisting medical conditions (other than the condition being studied) judged by the investigator to have worsened in severity or frequency or changed in character during the protocol-specified AE reporting period.

### Serious Adverse Events (SAE)

An AE should be classified as an SAE and reported as such, if it meets the following criteria:

- It results in death (i.e., the AE actually causes or leads to death).
- It is life threatening (i.e., the AE, in the view of the investigator, places the subject at immediate risk of death).
- It requires or prolongs inpatient hospitalization.
- It results in persistent or significant disability/incapacity (i.e., the AE results in substantial disruption of the subject’s ability to conduct normal life functions).
- It results in a congenital anomaly/birth defect in a neonate/infant born to a mother exposed to the investigational product.
- It is considered a significant medical event by the investigator based on medical judgment (e.g., may jeopardize the subject or may require medical/surgical intervention to prevent one of the outcomes listed above).
- It is considered sight-threatening by the investigator.

If a subject is hospitalized to undergo a medical or surgical procedure as a result of an AE, the event responsible for the procedure, not the procedure itself, should be recorded as the event.

Hospitalizations for the following reasons will not be recorded as SAEs:

- Hospitalization or prolonged hospitalization for diagnostic or elective surgical procedures for preexisting conditions.
- Hospitalization or prolonged hospitalization required to allow outcome measurement for the study.
- Hospitalization or prolonged hospitalization for scheduled therapy of the target disease of the study.

### Sight-threatening Events

An event is considered a sight-threatening and should be reported as an SAE if it meets one or more of the following criteria:

- It caused a decrease in visual acuity of ≥30 letters (compared with the last assessment of visual acuity at the last visit) lasting >1 hour.
- It caused a decrease in visual acuity to the level of Light Perception or worse lasting >1 hour.
- It required surgical intervention (e.g., conventional surgery, vitreous tap or biopsy with intravitreal injection of anti-infectives, or laser or retinal cryopexy with gas) to prevent permanent loss of sight.
- It is associated with severe intraocular inflammation (i.e., 4+ anterior chamber cell/flare or 4+ vitritis).
- In the opinion of the investigator it may require medical intervention to prevent permanent loss of sight.

### Life-threatening

Any adverse experience, for which the subject was at risk of death at the time of the event, is considered as life-threatening. It does not refer to an event which hypothetically might have caused death if it were more severe.

### Death

All deaths that occur during the protocol-specified AE reporting period, regardless of attribution, will be recorded and expeditiously reported to the Sponsor.

When recording a death, the event or condition that caused or contributed to the fatal outcome should be recorded as the AE event term.

## Adverse Event Assessment and Documentation

All subjects who have been exposed to the study treatment will be evaluated for adverse events. All adverse events, regardless of severity and whether they are ascribed to the study treatment, will be recorded using standard medical terminology. The investigator *must* assess (and record in the source documents and CRF) whether the event is related to the study device.

All adverse events will be evaluated beginning with onset, and evaluation will continue until resolution is noted, or until the investigator determines that the subject’s condition is stable, whichever is earlier. The investigator will take all appropriate and necessary therapeutic measures required for resolution of the adverse event. Any medication necessary for the treatment of an adverse event must be recorded on the concomitant medication case report form. If more than one distinct adverse event occurs, each event should be recorded separately.

All AEs will be characterized by the following criteria:

- Event Term
- Intensity or Severity
- Expectedness
- Relationship
- Outcome
- Treatment or Action Taken

## Adverse Event Terms

Whenever possible, use recognized medical terms when recording AEs. Do not use colloquialisms and/or abbreviations. Only one medical concept, preferably a diagnosis instead of individual symptoms, should be recorded as the event.

If known at the time of reporting, a diagnosis (i.e. disease or syndrome) should be recorded on the CRF rather than individual signs and symptoms (e.g., record congestive heart failure rather than dyspnea, rales, and cyanosis). However, if a constellation of signs and/or symptoms cannot be medically characterized as a single diagnosis or syndrome at the time of reporting, each individual event should be recorded as a separate AE. If a diagnosis is subsequently established, it should be reported as follow-up information. However, signs and symptoms that are considered unrelated to an encountered syndrome or disease should be recorded as individual AEs (e.g., if congestive heart failure and severe headache are observed at the same time, each events should be recorded as an individual AE).

Adverse events occurring secondary to other events (e.g., sequelae) should be identified by the primary cause; a "primary" event, if clearly identifiable, and generally represents the most accurate clinical term to record as the AE event term.

## Classification of Adverse Events by Intensity/Severity

All adverse events should be graded on a four-point scale (mild, moderate, marked, severe) for intensity/severity. Unless otherwise defined in the protocol or PSM, the definitions are as follows:

**Mild:** Transient discomfort; no medical intervention/therapy required and does not interfere with daily activities.

**Moderate:** Low level of discomfort or concern with mild to moderate limitation in daily activities; some assistance may be needed; minimal or no medical intervention/therapy required.

**Marked:** Considerable discomfort with limitation in daily activities, some assistance usually required; medical intervention/therapy usually required.

**Severe:** Extreme discomfort and limitation in daily activities, significant assistance required; ***significant*** medical intervention/therapy required.

There is a distinction between the severity and the seriousness of an adverse event. Severity is a measurement of intensity; thus, a severe reaction is not necessarily a serious adverse event (SAE). For example, a headache may be severe in intensity, but would not be serious unless it met one of the criteria for serious adverse events listed previously.

## Expectedness

All AEs related to the device or associated procedure will be evaluated as unexpected to occur.

- **Unexpected (unanticipated):** An adverse event is unexpected when the specificity or severity of an adverse event is not consistent with the applicable product information (i.e., Investigator’s Brochure or risk information described in the investigational plan/protocol and informed consent). Unexpected as defined here refers to an adverse event that has not been observed before.

## Relationship

The Principal Investigator (PI) will evaluate if the AE is related to the study device. Relationship is defined in the following manner:

**Not related:** Evidence indicates no plausible direct relationship to the study device, such that:

- A clinically plausible temporal sequence is inconsistent with the onset of the AE and device administration; and/or
- A causal relationship is considered biologically implausible.
- The AE can be attributed to concurrent/underlying illness, other drugs, or procedures.

**Related:** Evidence indicates a reasonable temporal sequence of the event with the study device administration exists, or that the association of the event with study device administration is unknown and the event is not reasonably supported by other conditions, such that:

- There is a clinically plausible time sequence between onset of the AE and study treatment administration; and/or
- There is a biologically plausible mechanism for study treatment causing or contributing to the AE; and
- The AE cannot be reasonably attributed to concurrent/underlying illness, other drugs, or procedures.

## Outcome

The clinical outcome of an AE will be characterized as follows:

- Resolved without sequelae
- Resolved with sequelae
- Ongoing (i.e. continuing at time of study discontinuation)
- Death

## Treatment or Action Taken

- None
- Surgical Intervention
- Medical Intervention
- Other

## Additional AE Definitions

### Definitions of Visual loss

- ***Moderate visual loss*** will be defined as a decrease of ≥3 lines (≥15 letters) from baseline for at least 1 hour.
- ***Significant visual loss*** will be defined as a decrease of ≥6 lines (≥30 letters) from baseline for at least 1 hour.

# STUDY DISCONTINUATION

The Investigator has the right to terminate this study at any time. Reasons for terminating the study may include, but are not limited to, the following:

- The incidence or severity of adverse events in this or other studies indicates a potential health hazard to subjects.
- Subject enrollment is unsatisfactory.
- Data recording is inaccurate or incomplete.

# STATISTICS

## Efficacy Endpoints

The primary efficacy outcome of this trial is the change in retinal sensitivity on customized microperimetry.

**Other additional secondary outcomes will include the following at 12 weeks:**

Change in mean VA

Change in OCT features in the treated area

Change in total hypofluorescence in the treated area

## Statistical Methodology and Sample Size

A sample size of 16 eyes has a greater than 80% power to identify a variation of 1.5 decibels in macular sensitivity between pre and post laser treatment assessments, with an estimated standard deviation of the change outcome of 2.0 and an alpha error of 0.05. Allowing an additional 20% of the estimated sample size in order to counter possible withdrawn patients, we estimated that 20 eyes would be required in our series.

Statistical analysis on retinal sensitivity before and after treatment will be carried out within group of pathology.

The Repeated Measures Analysis of Variance will be carried out to verify the effect of treatment.

## Safety

Descriptive analysis of listed Adverse Events.

# PROTOCOL DEVIATIONS / AMENDMENTS

Investigators should make every attempt to not deviate from the protocol. Deviations can ultimately affect the scientific soundness of the protocol, as well as the rights, safety and welfare of the subject.

# ETHICAL PRECEPTS

The Investigator will ensure that the clinical study is conducted in accordance with good clinical practice and all regulatory and institutional requirements, including those for subject privacy, informed consent, Ethics Committee or Ministry of Health approval and record retention, and with the Declaration of Helsinki (revised October 2008).

The Investigator must observe the requirements of the appropriate regulatory body by obtaining written informed consent. The study informed consent form must be obtained prior to the initiation of any study procedures. The subject (or the subject's legally authorized representative) must be allowed sufficient time to thoroughly read (or have explained), the informed consent form. The Investigator should answer any questions that the subject/representative might have. If the subject agrees to participate in the study, the subject/representative must sign both copies of the informed consent form. The witness and the Investigator must also sign both copies of the informed consent form. One copy of the informed consent form should be given to the subject/representative. If applicable, it will be provided in a certified translation of the local language. The date of the subject’s signature on the informed consent form should be noted in the subject’s medical chart to document that informed consent was obtained prior to initiating any study procedures.

Signed consent forms must remain in each subject’s study file and must be available for verification by study monitors at any time.

**REFERENCES**

1. Miller J.W. Age-related macular degeneration revisited—Piecing the puzzle: The LXIX edward jackson memorial lecture. Am. J. Ophthalmol. 2013;155

2. Ferris F.L., III, Fine S.L., Hyman L. Age-related macular degeneration and blindness due to neovascular maculopathy. Arch. Ophthalmol. 1984;102:1640–1642.

3. Green WR, Enger C. Age-related macular degeneration histopathologic studies. The 1992 Lorenz E. Zimmerman Lecture. Ophthalmology 1993;100:1519–35.

4. Zweifel SA, Spaide RF, Curcio CA, et al. Reticular pseudodrusen are subretinal drusenoid deposits. Ophthalmology 2010; 117:303–12.

5. Finger RP et al. Reticular Pseudodrusen and Their Association with Age-Related Macular Degeneration: The Melbourne Collaborative Cohort Study. Ophthalmology. 2015 Dec 8. pii: S0161-6420(15)01216-6.

6. Nowak JZ. Age-related macular degeneration (AMD): pathogenesis and therapy. Pharmacol Rep. 2006;58:353-363.

7. Sunness JS, Applegate CA, Bressler NM, Hawkins BS. Designing clinical trials for age- related geographic atrophy of the macula. Retina. 2007;27:204-210.

8. Sunness JS, Rubin GS, Applegate CA, Bressler NM, Marsh MJ, Hawkins BS, et al. Visual function abnormalities and prognosis in eyes with age-related geographic atrophy of the macula and good visual acuity. Ophthalmol. 1997;104:1677-1691.

9. Sunness JS, Rubin GS, Broman A, Applegate CA, Bressler NM, Hawkins BS. Low luminance visual dysfunction as a predictor of subsequent visual acuity loss from geographic atrophy in age related macular degeneration. Ophthalmol. 2008;115(9):1480-1488, 1488.el-2.

10. American Academy of Ophthalmology Retina/Vitreous Panel. Preferred Practice Pattern®Guidelines. Age-Related Macular Degeneration. San Francisco, CA: American Academy of Ophthalmology; 2015.

11. Brown DM, Kaiser PK, Michels M, et al, ANCHOR Study Group. Ranibizumab versus verteporfin for neovascular age-related macular degeneration. N Engl J Med 2006;355:1432-44.

12. Rosenfeld PJ, Brown DM, Heier JS, et al, MARINA Study Group. Ranibizumab for neovascular age-related macular degeneration. N Engl J Med 2006;355:1419-31.

13. Heier JS, Brown DM, Chong V, et al, VIEW 1 and VIEW 2 Study Groups. Intravitreal aflibercept (VEGF trap-eye) in wet age-related macular degeneration. Ophthalmology 2012;119:2537-48.

14. Martin DF, Maguire MG, Ying GS, et al, Comparison of Age-related Macular Degeneration Treatments Trials (CATT) Research Group. Ranibizumab and bevacizumab for neovascular age-related macular degeneration. N Engl J Med 2011;364:1897-908.

15. Luttrull JK, et al, Laser resensitization of medically unresponsive neovascular agerelated macular degeneration. Retina 2015; 35:1184–1194.

16. Bressler NM, Treatment of Age-Related Macular Degeneration with Photodynamic Therapy (TAP) Study Group. Photodynamic therapy of subfoveal choroidal neovascularization in age-related macular degeneration with verteporfin: two-year results of 2 randomized clinical trials-TAP report 2. Arch Ophthalmol 2001;119:198-207.

17. Barbazetto I, Burdan A, Bressler NM, et al. Photodynamic therapy of subfoveal choroidal neovascularization with verteporfin: fluorescein angiographic guidelines for evaluation and treatment--TAP and VIP report number 2. Arch Ophthalmol 2003;121:1253-68.

18. Macular Photocoagulation Study Group. Argon laser photocoagulation for neovascular maculopathy: five-year results from randomized clinical trials. Arch Ophthalmol 1991;109:1109-14.

19. Dorin G. Subthreshold and micropulse diode laser photocoagulation. Semin Ophthalmol 2003;18:147-53.

20. Luttrull JK, Musch DC, Mainster MA. Subthreshold diode micropulse photocoagulation for the treatment of clinically significant diabetic macular oedema. Br J Ophthalmol 2005; 89:74–80.

21. Parodi MB, Spasse S, Iacono P, et al. Subthreshold grid laser treatment of macular edema secondary to branch retinal vein occlusion with micropulse infrared (810 nanometer) diode laser. Ophthalmology 2006;113:2237-42.

22. Chen SN, Hwang JF, Tseng LF. Subthreshold diode micropulse photocoagulation for the treatment of chronic central serous chorioretinopathy with juxtafoveal leakage. Ophthalmology 2008;115:2229-34.

23. Luttrull JK, Sinclair SH. Safety of transfoveal subthreshold diode micropulse laser (SDM) for fovea-involving diabetic macular edema in eyes with good visual acuity. Retina 2014; 34:2010–2020.

24. Luttrull JK, Chang DB, Margolis BW. LASER RESENSITIZATION OF MEDICALLY UNRESPONSIVE NEOVASCULAR AGE-RELATED MACULAR DEGENERATION: Efficacy and Implications. Retina 2015;35:1184-94.

25. Luttrull JK, Musch DC, Mainster MA. Subthreshold diode micropulse photocoagulation for the treatment of clinically significant diabetic macular oedema. Br J Ophthalmol 2005; 89:74–80.

26. Luttrull JK, Sinclair SH. Safety of transfoveal subthreshold diode micropulse laser (SDM) for fovea-involving diabetic macular edema in eyes with good visual acuity. Retina 2014; 34:2010–2020.

27. Vujosevic S, et al. subthreshold micropulse yellow laser versus subthreshold micropulse infrared laser in center-involving diabetic macular edema. Retina 2015; 35:1594–1603.

28. Kenichiro B, et al. Effect of subthreshold infrared laser treatment for Drusen regression on macular autofluorescence in patients with age-related macular degeneration. Retina 2005; 25: 981-988.

29. Virgili G, et al. Laser treatment of drusen to prevent progression to advanced age-related macular degeneration. Cochrane Database Syst Rev 2015; Issue 10.
